# Transforming Cancer Care: A Narrative Review on Leveraging Artificial Intelligence to Advance Immunotherapy in Underserved Communities

**DOI:** 10.3390/jcm14155346

**Published:** 2025-07-29

**Authors:** Victor M. Vasquez, Molly McCabe, Jack C. McKee, Sharon Siby, Usman Hussain, Farah Faizuddin, Aadil Sheikh, Thien Nguyen, Ghislaine Mayer, Jennifer Grier, Subramanian Dhandayuthapani, Shrikanth S. Gadad, Jessica Chacon

**Affiliations:** 1 Foster School of Medicine, Texas Tech University Health Sciences Center El Paso, El Paso, TX 79905, USA; victor.m.vasquez@ttuhsc.edu (V.M.V.J.); momccabe@ttuhsc.edu (M.M.); jack.c.mckee@ttuhsc.edu (J.C.M.); sharon.siby@ttuhsc.edu (S.S.); uhussain@ttuhsc.edu (U.H.); fafaizud@ttuhsc.edu (F.F.); aadishei@ttuhsc.edu (A.S.); damayer@ttuhsc.edu (G.M.); s.dhandayuthapani@ttuhsc.edu (S.D.); 2 School of Medicine Greenville, University of South Carolina, Greenville, SC 29605, USA; nguyentp@email.sc.edu (T.N.); jgrier@greenvillemed.sc.edu (J.G.); 3 Center of Emphasis in Infectious Diseases, L. Frederick Francis Graduate School of Biomedical Sciences, Texas Tech University Health Sciences Center El Paso, El Paso, TX 79905, USA; 4 South Texas Center of Excellence in Cancer Research, Department of Medicine and Oncology, The University of Texas Rio Grande Valley School of Medicine, McAllen, TX 78504, USA; 5 Center of Emphasis in Cancer, Department of Molecular and Translational Medicine, Texas Tech University Health Sciences Center El Paso, El Paso, TX 79905, USA

**Keywords:** cancer immunotherapy, health disparities, artificial intelligence, social determinants of health (SDOH), Predictive Modeling

## Abstract

**Purpose**: Cancer immunotherapy has transformed oncology, but underserved populations face persistent disparities in access and outcomes. This review explores how artificial intelligence (AI) can help mitigate these barriers. **Methods**: We conducted a narrative review based on peer-reviewed literature selected for relevance to artificial intelligence, cancer immunotherapy, and healthcare challenges, without restrictions on publication date. We searched three major electronic databases: PubMed, IEEE Xplore, and arXiv, covering both biomedical and computational literature. The search included publications from January 2015 through April 2024 to capture contemporary developments in AI and cancer immunotherapy. **Results**: AI tools such as machine learning, natural language processing, and predictive analytics can enhance early detection, personalize treatment, and improve clinical trial representation for historically underrepresented populations. Additionally, AI-driven solutions can aid in managing side effects, expanding telehealth, and addressing social determinants of health (SDOH). However, algorithmic bias, privacy concerns, and data diversity remain major challenges. **Conclusions**: With intentional design and implementation, AI holds the potential to reduce disparities in cancer immunotherapy and promote more inclusive oncology care. Future efforts must focus on ethical deployment, inclusive data collection, and interdisciplinary collaboration.

## 1. Introduction

### 1.1. A Paradigm Shift in Oncology

Cancer immunotherapy has revolutionized the landscape of oncology, marking a fundamental shift in how malignancies are treated. Unlike the conventional approaches that have traditionally been at the forefront in the fight against cancer, such as chemotherapy and radiation, which indiscriminately target both healthy and cancerous cells—often leading to severe side effects—immunotherapy leverages the body’s own immune defenses to precisely identify and destroy cancer cells [1,2]. Breakthroughs in treatments, such as immune checkpoint inhibitors, Chimeric Antigen Receptor (CAR)-T cell therapy, and cancer vaccines, have significantly improved survival rates and long-term prognoses for patients battling various forms of cancer [3,4,5]. Notably, these therapies have shown remarkable efficacy in animal models once deemed highly challenging, such as metastatic renal cell carcinoma and specific types of brain tumors [6,7]. However, despite these advancements, widespread access to immunotherapy remains a pressing issue. Many underserved populations continue to face disparities in treatment availability, effectiveness, and outcomes [8]. Geographic, economic, and systemic obstacles prevent numerous patients from receiving cutting-edge care, ultimately limiting the full potential of these groundbreaking therapies [8].

### 1.2. Disparities in Access to Cancer Immunotherapy

Addressing health disparities in cancer immunotherapy remains a significant challenge in modern oncology. Socioeconomic status (SES) and social determinants of health (SDOH) are key contributors to disparities in access to cancer immunotherapy and treatment effectiveness [8]. Individuals from lower-income backgrounds are less likely to receive advanced cancer treatment, including immunotherapy, due to barriers such as limited access to specialized care and financial constraints [8].

These disparities arise from multiple factors, such as limited access to specialized cancer centers, financial constraints, and underrepresentation in clinical trials. Beyond structural barriers, implicit biases in healthcare delivery and a lack of culturally competent care may further widen these gaps, leading to delayed diagnoses and less effective treatment plans [9,10]. Addressing these challenges requires strengthening healthcare infrastructure, improving provider training on fair care, and ensuring access to high-quality cancer services across populations. Closing these gaps is essential to ensuring access to cancer care and improving outcomes for all patients, regardless of socioeconomic or demographic background.

### 1.3. The Promise of Artificial Intelligence in Reducing Disparities

**Figure 1:**
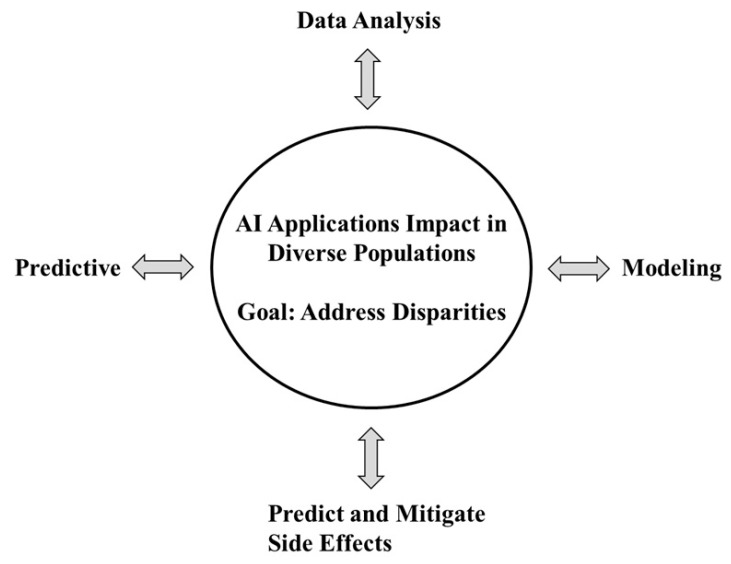
AI Applications and Predictive Models in Addressing Disparities and Adverse Event Management. Figure 1 illustrates key applications of artificial intelligence in healthcare—Data Analysis, Predictive Modeling, Clinical Trials, and Side Effect Mitigation—and their collective impact on diverse populations. Positioned at the center of the figure, the outcomes emphasize AI’s potential to reduce health disparities and proactively manage adverse events through inclusive, data-driven strategies.

The integration of artificial intelligence (AI) into healthcare systems represents a pivotal advancement in our pursuit of more equitable, accessible, and patient-centered care. AI has emerged as a transformative tool with the potential to reduce disparities in cancer care [11]. Figure 1 highlights the interconnected roles of AI in healthcare through four primary applications: Data Analysis, Predictive Modeling, Clinical Trials, and Side Effect Mitigation. These components surround a central focus on improving outcomes in diverse populations by addressing disparities and enhancing the management of adverse events. The circular layout emphasizes the continuous, integrated nature of AI-driven solutions in promoting personalized care.

Through machine learning, natural language processing, and predictive analytics, AI can enhance early detection, refine treatment selection, and improve patient monitoring [12,13]. Table 1 provides a comparative summary of key AI technologies and models used in cancer immunotherapy, detailing their primary applications, core features, performance metrics, and implications for health inclusivity. Tools and models include Natural Language Processing, Machine Learning, Computer Vision, and specialized platforms such as PathAI and DeepSurv. The column on disparity impact highlights how these technologies can enhance accessibility, personalize care, or mitigate bias—while also noting the risks of reinforcing fairness if diverse datasets and transparent implementation are not prioritized. By analyzing vast datasets, AI-driven models can identify treatment response patterns, personalize immunotherapy regimens, and anticipate adverse effects—ultimately leading to more effective and inclusive cancer treatment [14,15]. Additionally, AI-powered clinical decision support systems assist oncologists in navigating complex treatment landscapes, ensuring that therapies are tailored to each patient’s unique genetic and molecular profile [16]. Beyond treatment optimization, AI can also help increase representation of underserved populations in research, leading to a more comprehensive understanding of immunotherapy’s efficacy across diverse demographic groups [17,18]. As illustrated by the successful deployment of AI tools in areas such as diabetic foot ulcer monitoring and retinal screening for diabetic eye disease, these technologies are already making tangible improvements in clinical outcomes, care coordination, and access—especially in resource-limited communities.

### 1.4. Expanding Access Through AI-Driven Care Models

All in all, integrating AI into clinical workflows can seemingly offer a pathway to reducing disparities and making immunotherapy more accessible. By providing real-time clinical decision support, enabling remote diagnostics, and facilitating early detection of complications, AI is helping shift the paradigm from reactive to proactive care. Moreover, AI’s role in telemedicine and remote patient monitoring can help bridge geographic barriers, providing continuous care to patients who may lack access to major cancer centers. By enhancing treatment adherence and improving survival outcomes, AI stands as a critical asset in the effort to achieve access to cancer care for all.

In addition, AI-powered decision support tools can assist clinicians in community and resource-limited settings by offering real-time guidance on immunotherapy eligibility, biomarker interpretation, and management of immune-related adverse events. These tools democratize specialized knowledge, empowering non-specialists to deliver high-quality care in regions where oncology subspecialists are scarce. Furthermore, AI can streamline patient triage and referral systems, ensuring that high-risk individuals are identified earlier and connected to appropriate care pathways [19,20]. When integrated with mobile health platforms and multilingual interfaces, these technologies can further enhance outreach to linguistically and culturally diverse populations, ultimately sup-porting a more accessible oncology landscape.

### 1.5. Narrative Review Approach: Literature Search and Selection Strategy

This narrative review draws upon peer-reviewed literature and key sources selected for relevance to artificial intelligence, cancer immunotherapy, and healthcare disparities. Sources were identified through author expertise and iterative exploration of the existing literature, without restriction on publication date.

To enhance the transparency and rigor of this narrative review, we employed a structured literature search and screening process to identify relevant studies addressing the intersection of artificial intelligence (AI), cancer immunotherapy, and healthcare disparities, particularly in underserved populations.

We searched three major electronic databases: PubMed, IEEE Xplore, and arXiv, covering both biomedical and computational literature. The search included publications from January 2015 through April 2024, to capture contemporary developments in AI and cancer immunotherapy.

Keywords and Boolean operators used in various combinations included:

- artificial intelligence” OR “machine learning” OR “deep learning”
- cancer immunotherapy” OR “immune checkpoint inhibitors” OR “tumor microenvironment”
- health disparities” OR “underserved populations” OR “minority health” OR “equity”

Search queries were adapted to the syntax of each database, and filters were applied to select English-language, peer-reviewed, full-text articles.

Inclusion criteria: Studies were included if they met the following criteria:

- Focused on AI applications in cancer immunotherapy (e.g., biomarker discovery, outcome prediction, treatment optimization).
- Reported outcomes related to model performance, clinical utility, or patient impact.
- Discussed or analyzed demographic variables, population diversity, or implications for underserved communities.
- Published in a peer-reviewed journal or reputable preprint repository.

Exclusion criteria:

- Focused solely on traditional (non-immunotherapy) cancer treatments.
- Lacked sufficient methodological detail or performance metrics.
- Were editorials, opinion pieces, or conference abstracts without data.

## 2. Disparities in Cancer Immunotherapy

### 2.1. Disparities in Access and Outcomes

Cancer immunotherapy has transformed the treatment for many malignancies, yet significant disparities persist in the access and outcomes among underrepresented populations. Research has consistently shown that certain population groups experience lower rates of immunotherapy utilization, even though available evidence suggests they may respond similarly to these treatments. [8]. This discrepancy arises from many factors such as socioeconomic stratum, insurance coverage, and low clinical trial enrollment of minorities [21]. SES and SDOH further exacerbate these inequities this influencing the availability, affordability, and effectiveness of immunotherapy of communities [8].

### 2.2. Cognitive Influences on Clinical Decision-Making

Variability in clinical decisions can arise not only from system-level factors but also from individual-level cognitive influences. Subtle, unconscious mental shortcuts—often referred to as implicit biases—can unintentionally shape provider judgment, communication, and care delivery. For example, some studies have shown differences in the likelihood of recommending screening procedures based on non-clinical characteristics [22]. Research suggests a negative correlation between higher levels of implicit bias and the overall quality of clinical interactions, reinforcing the need for strategies that support more standardized, data-driven decision-making. AI-assisted clinical tools, particularly when integrated into electronic health records and supported by diverse training data, may offer potential to reduce this variability by grounding decisions in consistently applied evidence-based algorithms.

### 2.3. Geographic Distribution of the Healthcare Workforce

A longstanding challenge in U.S. healthcare is the uneven geographic distribution of healthcare providers. While approximately 20% of the U.S. population lives in rural areas, only about 10% of physicians practice there [22]. This discrepancy is expected to widen, with rural regions projected to face a shortfall of more than 20,000 physicians by 2025 [23]. Contributing factors include professional isolation, limited access to specialized equipment and support staff, fewer training opportunities, and a lack of long-term incentives to practice in remote or under-resourced settings [22]. This maldistribution contributes to variation in care delivery capacity and timeliness across regions. AI-enhanced platforms may help mitigate this by supporting remote consultations, diagnostic triage, and workflow optimization—thereby extending the reach of care teams without requiring constant physical presence.

### 2.4. Limited Access to Specialized Services in Medically Isolated Areas

Beyond rural regions, some areas have been characterized as “medical deserts,” defined as locations with limited availability of healthcare professionals and critical services [22]. Obstetric care provides a striking example: while urban residents typically live within 8 miles of such facilities, patients in medically isolated areas may have to travel 28 miles or more to access care [22]. Approximately 2 million individuals of childbearing age live in areas fitting this description, and geographic barriers have been associated with increased risk of complications during pregnancy and the postpartum period. Innovations in AI-driven remote diagnostics and triage systems may support timely interventions in these areas, helping to reduce travel-related delays and facilitate earlier detection of clinical concerns.

### 2.5. Transportation Barriers and Continuity of Care

Transportation is a common obstacle in maintaining continuity of care, especially for individuals managing chronic conditions. Repeated visits are often required for disease monitoring, medication management, and follow-up evaluations. Studies have shown that patients with a driver’s license attend more than twice as many healthcare appointments compared to those without one, and having access to a family member or friend for transportation also increases healthcare utilization [22,24]. AI-integrated mobile health (mHealth) platforms and remote monitoring tools may provide alternatives for patients facing transportation barriers. These solutions allow for aspects of care to be managed virtually, enabling healthcare teams to maintain regular contact with patients and intervene when necessary.

### 2.6. Gaps in Representation in Clinical Trials and Real-World Data

Despite broad consensus on the importance of inclusive research, gaps in representation persist across many clinical trials and observational studies. Accurate representation ensures that resulting data and treatment guidelines are applicable to the full spectrum of patients seen in real-world practice [25]. However, populations such as older adults, individuals with comorbidities, and women remain underrepresented in numerous therapeutic areas, including oncology, cardiology, and infectious disease [22]. For instance, women make up only 40.2% of participants in cancer treatment trials and just 26.5% in prevention trials [26,27]. Older adults are frequently excluded due to polypharmacy or complex medical histories, despite their high disease burden. These gaps may limit the applicability of findings and influence treatment outcomes if interventions are less tailored to real-world populations.

AI and machine learning can help address some of these limitations by analyzing large, heterogeneous datasets from clinical practice to complement trial data. Real-world evidence platforms powered by AI may improve understanding of treatment effects across diverse patient profiles and help guide more personalized treatment recommendations—supporting expanded access to therapies such as immunotherapy in varied clinical contexts.

## 3. The Role of AI in Addressing Disparities

### 3.1. AI in Analyzing SES and SDOH

AI is revolutionizing the analysis of SES and SDOH by leveraging large-scale datasets, including electronic health records and public health databases. Machine learning algorithms can process complex, multidimensional data to identify hidden patterns that influence healthcare disparities. AI-driven insights can elucidate the impact of income, education, geographic location, and access to healthcare services on immunotherapy outcomes. These outcomes can provide actionable intelligence to inform changes, resource allocation, and targeted interventions aimed at reducing health disparities.

Many strategies can be leveraged by AI to expand its utility in identifying and interpreting SDOH and SES factors through natural language processing (NLP), deep learning, and structured data mining. NLP methods have been demonstrated to effectively isolate factors encompassing SDOH such as employment status, social support and housing instability from electronic health records in emergency settings [28]. Despite the efficacy of AI in parsing and extracting information relevant to health disparities in large datasets, they can also inadvertently reinforce negative biases in performance. Machine learning algorithms that are trained on incomplete electronic health records demonstrated lower performance in predicting asthma adverse effects in low-SES children due to a lack of robust data within the records [29].

Overall, AI offers a powerful toolkit to analyze SES and SDOH, making it possible to identify disparities, predict health outcomes, and provide guidance on medical interventions. Future work should focus on harmonizing data standards, ensuring representativeness across populations, and integrating AI outputs into clinical workflows to enable socially informed, precision healthcare.

### 3.2. Predictive Modeling for Side Effects

AI-driven predictive models play a crucial role in identifying and mitigating adverse effects associated with immunotherapy. By analyzing patient-specific factors, treatment histories, and real-world evidence, AI can forecast potential side effects and guide clinicians in preemptive intervention. This capability is particularly valuable for underserved populations, who often experience higher toxicity rates due to comorbidities and limited access to specialized care.

Studies have shown that neural network models can been utilized to predict dermatological immune adverse events due from PD-L1 therapy by considering variables such as tumor type, treatment drug, patient age, autoimmune history, and various laboratory values [22]. These models facilitate the early identification of patients at risk, allowing for timely management strategies that can mitigate the severity of side effects and improve adherence to treatment protocols [22].

AI-powered tools can assist in customizing supportive care strategies, ensuring timely management of complications and improving adherence to treatment regimens. Moreover, AI can integrate genetic, environmental, and behavioral data to enhance precision in predicting and managing immunotherapy-related toxicities. Despite these advancements, the integration of AI models into clinical workflows requires careful consideration of data privacy, algorithm transparency, and the need for standardized validation across diverse patient populations.

### 3.3. Risk Stratification and Tailored Interventions

AI enables robust risk stratification by classifying patients based on their likelihood of experiencing poor immunotherapy outcomes. Machine learning models can incorporate diverse patient data, including biomarkers, comorbid conditions, and SDOH, to create individualized risk profiles. This stratification allows healthcare providers to deploy targeted interventions, optimizing treatment pathways and resource allocation. AI models that merge traditional data sources with SDOH indicators can more accurately identify high-cost members compared to conventional risk scoring systems. This integration allows providers to effectively allocate resources and implement interventions that address both medical and social factors influencing patient outcomes [30]. Other examples of AI-driven interventions include remote monitoring programs for high-risk patients, AI-powered chatbots for real-time patient engagement, and decision-support systems that recommend alternative treatment options for vulnerable populations [22]. These tools facilitate real-time patient engagement and continuous monitoring, ensuring timely responses to emerging health issues and improving overall care quality [22]. These innovations help bridge care gaps and improve rational, AI-driven cancer care in immunotherapy access and efficacy.

### 3.4. Neoantigen Identification Using AI in Underrepresented Populations

AI-driven approaches to neoantigen identification have the potential to enhance immunotherapy efficacy in underrepresented populations. Traditional cancer research has often overlooked diverse genetic backgrounds, leading to disparities in treatment outcomes. AI can analyze vast genomic datasets to identify tumor-specific neoantigens unique to different racial and ethnic groups, improving vaccine and T-cell therapy designs [31,32,33]. Several initiatives leverage AI for this purpose, such as deep learning models that predict neoantigen immunogenicity and AI-powered pipelines that integrate multi-omics data for personalized cancer immunotherapy [31,32,33]. By prioritizing diversity in AI model training and validation, researchers can drive more inclusive and effective immunotherapy strategies, reducing the gap in cancer treatment disparities.

## 4. Applications of AI to Improve Care Quality

### 4.1. Natural Language Processing

With the exponential growth of NLP across industries, healthcare is expected to benefit substantially. The crucial question is not whether NLP will impact healthcare, but rather when and how it can best enhance the accessibility, efficiency, and affordability of care for patients impacted by cancer. AI-based tools, like ChatGPT, have already proven valuable in medical education, aiding students and clinicians alike [34]. However, language barriers and health literacy continue to hinder patient satisfaction, quality of care, and safety [35]. There is significant potential for AI tool to improve physician communication with patients and patient education, particularly when language barriers exist or for patients with limited health literacy, although challenges remain in how to effectively employ AI tools in the clinical environment [35,36].

Despite promising applications, significant ethical and practical concerns about AI’s role in healthcare persist, underscoring the need for further research to explore responsible use [34,36,37,38]. One significant area of opportunity for NLP in healthcare is the capture of doctor-patient conversations for documentation [39], which has become more feasible due to recent AI advancements [40]. For instance, a standardized method for documenting patient care using AI has been developed recently by Paterson Health Technology Institution [41]. NLP can capture and record the breadth of information shared by the physician during a patient appointment as well as any patient questions and the resulting answers. For a cancer patient, NLP-assisted documentation can allow the provider to spend more time focused on communicating treatment options or outcomes and risks with the patient. Furthermore, the use of NLP in patient-provider communication has been shown to reduce physician burnout related to documentation [42,43]. While current AI capture methods for clinical notes remain imperfect [44], the available tools and accuracy continue to improve and can enhance the clinical experience for both the patient and the provider.

Furthermore, these AI NLP technologies can be employed to enhance patient education and understanding of clinical information. In our current health literacy education system, passive one-way information flow is common, however it does not provide a means for ensuring patient understanding. One potential application for Generative AI in the improvement of clinical patient education is in the form of NLP-generated Patient Understanding Assessments. AI tools can create a summary from the AI-generated clinical notes at an appropriate level of health literacy for the patient and then produce questions to assess patients’ understanding of the health information discussed. This AI NLP-generated assessment of patient education can highlight essential clinical information in an accessible manner for the patient and will allow for evaluation of patient understanding. These assessments can be administered prior to the patient leaving the clinic and can be conducted by medical assistants or nurses. Clinical staff can provide immediate clarification of general health concepts or signal to the provider if further discussion is needed in order to ensure that the patient is well informed about their care. The use of NLP patient understanding assessments may provide the means to confirm or enhance patient understanding as well as help physicians identify areas in need of additional explanation. AI has the potential to synthesize and convey complex health concepts effectively and this can prove especially helpful in combination with translation capabilities. Consequently, AI could help ensure that underrepresented patients, particularly immigrants, received accessible information about their health, cancer risks, and treatment processes in their preferred language and educational level. The more patients understand their disease and health, the better adherence and positive outcomes can be realized [45].

### 4.2. Machine Learning and Real Word Evidence

Recruiting participants for clinical trials remains an ongoing challenge for researchers and physicians, particularly due to underrepresentation of minority populations, patient retention issues, and logistical hurdles. Fortunately, the National Institutes of Health has recently developed an AI algorithm to streamline the recruitment process [46,47]. This algorithm can help identify suitable clinical trials for volunteers based on their specific profiles.

Additionally, AI has the potential to improve participant retention, especially within minority populations, who often juggle multiple responsibilities. Loss of follow-up often occurs due to difficulties in scheduling appointments or making phone calls. To mitigate this issue, AI-driven solutions such as chatbots would enable participants to conveniently “check in” at any time without the need for appointments or phone calls.

SDOH also plays a significant role in the health outcomes of diverse populations. AI has the potential to uncover gaps in our current understanding of how SDOH can influence health outcomes across different populations [48]. Furthermore, AI’s capacity to process vast amounts of data simultaneously surpasses human abilities, enabling more efficient and comprehensive analysis [49]. This powerful technology may contribute to addressing health disparities and improving outcomes for groups by illuminating the connections between social factors and their health, particularly in regard to cancer therapeutics and immunotherapy.

## 5. Ethical Considerations and Challenges

### 5.1. Algorithmic Bias

An algorithm is a set of instructions written to guide a computer towards desired outputs given certain inputs. We write algorithms to tell machines how to learn. Artificial intelligence can be allowed to learn from sets of labeled data, sets of unlabeled data, trial and error with rewards, or vast expanses of free data imposed on networks designed after the human brain which are termed “neural networks.” These learning styles can be combined in different ways, and the outcome is ideally a computer system that can execute functions and provide insights faster and at a larger scale than can be performed by individuals [50].

Many subcategories of AI, such as large language models, learn based on what we present them with. When we create algorithms to direct interactions with datasets, we often neglect to control for our own blind spots. The data we have is also biased, for the reasons previously mentioned—lack of recruitment to clinical trials or studies focused on issues relevant to these groups, among them. Even when programs are working exactly as they are designed to, there is no way for the machine to recognize the impact of its calculations on the social fabric—namely, when it is recreating and reinforcing barriers to patient treatment. Data revealing significant disparities in health outcomes based on patients’ financial security must be interpreted with care. These differences often reflect the complex and cumulative impact of social determinants of health—such as housing stability, access to nutritious food, and environmental exposures—rather than poverty itself acting as a direct biological factor. Treating SES as a clinical variable without accounting for these underlying burdens risks oversimplifying its influence on health.

A notable example comes from a group of researchers at a Chicago hospital who sought to develop a predictive model for hospital length of stay to inform resource allocation. While their goal was to enhance operational efficiency by identifying patients most likely to be discharged early, they discovered that the most powerful predictor was the patient’s zip code [51,52]. Those residing in higher-income, better-resourced neighborhoods tended to have shorter hospital stays. Although incorporating this variable would have improved the model’s predictive accuracy and potentially reduced costs, the researchers chose not to proceed. They recognized that doing so could unintentionally reinforce existing disparities by prioritizing care for individuals already advantaged by their social context.

This case underscores the ethical responsibility of healthcare data scientists and clinicians to critically assess how predictive models are designed and applied. In doing so, they can help ensure that technological innovation supports rational, AI-driven care, rather than inadvertently undermining it [53].

If we attempt to create AI systems that direct underserved populations to care, even for something as genetically linked as cancer, how can we train our programs to recommend effective and timely treatments when the data show underserved patients and patients of lower socioeconomic status receiving fewer and less aggressive treatments, later in their disease course? Despite mistakes such as those made at Amazon, the use of AI in all sectors continues to explode. Even with good intentions, those interested in AI’s potential in streamlining healthcare provision run the risk of missing those we wish to serve. Purposefully consulting target populations in the creation of the machine learning model may help but not eliminate the risk of using a “black box” to guide decisions.

### 5.2. Privacy

The purest form of medical decision-making may be personalized medicine. The Precision Medicine Initiative defines personalized medicine as “an emerging approach for disease treatment and prevention that takes into account individual variability in genes, environment, and lifestyle for each person” [54]. We often herald AI as the next best tool for turning swaths of personal data into such unique recommendations. It is perhaps not surprising that the greatest use for AI in the medical industry thus far is cancer care, where each individual’s tumor represents an enormous complexity of data.

Ensuring patient privacy is essential to the longevity and success of AI models in cancer care, exactly because gathering the amount of genetic and personal information that would be needed to run the programs poses a threat to that privacy. Unfortunately, AI models that are simple and draw from large pools of data tend to perform better than complex models managing smaller datasets [54]. Deep learning machines such as ones that utilize neural networks described in the previous section require almost unlimited access to data to achieve their greatest predictive accuracy [55]. Despite degrees of uncertainty involved in its application, the best way forward from the perspective of computer and biomedical scientists in cancer research is to gather enormous quantities of patient data [54].

Without constant and perhaps universal approval for free access to Protected Health Information, AI’s run the risk of becoming outdated and failing to recommend the best care available. That being said, the very nature of the Health Insurance Portability and Accountability Act requires permission for the release of PHI and the option to rescind this permission at any time except under special circumstances [56]. The Health Insurance Portability and Accountability Act is our primary law protecting healthcare clients from unacceptable usage of their data, and addendums specific to AI should be a priority. As a society we must decide whether information that is so specific can truly be depersonalized and what protections have to exist to make its use acceptable. Since the primary goal of using AI is to make healthcare more efficient, deciding acceptable parameters should ideally fall to those most vulnerable and liable to suffer negative consequences of having their information taken without appropriate consent.

The speed and ease of genomic data collection is guaranteed to increase in the coming years, and the transition by and large to computer-based record keeping makes obtaining the data simple for a machine learning model if it is given access. Open-source and clinical trial collaborations, among others, will enhance this process. Before implementing AI on a wide scale, we must consider the benefits and harms, to amassing genetic and environmental data from patients. We also have to employ objective prospective validation studies to assess whether the deep learning models are closing the gaps in medical access and improving outcomes for the underserved as they portend to [57].

### 5.3. Need for Transparency

AI programs do not fall neatly into any prior categories of entity under medical law. The learned intermediary doctrine of Tort law protects drug or medical product manufacturers from direct lawsuit from patients under the assertion that the doctor is the “end consumer” of the product with the best understanding of its effects. The physician, as the end consumer, is held responsible for ensuring transparency about the product’s benefits and harms. AI programs are products used in patient care, but who is the end consumer of a product that grows and learns on its own? This “black box” concept makes it difficult to hold both physicians and coders legally accountable for the recommendations it may make [58]. Giving the AI system less autonomy and more oversight negate some of its time and cost-saving benefits. Increasing oversight may even compromise the basic purpose and utility of an unlimited search engine like ChatGPT. Proposals for increasing accountability include and are not limited to distributing the cost of harms suffered among all the AI’s implementers, which would in theory promote the physicians and staff’s interest in becoming the most educated end-consumers of the product possible. This may result in improved transparency from physicians sharing information about AI with the patient and the pros and cons of using AI recommendation in the same way they would do for a tablet of Aspirin.

As we move forward with the implementation of AI in healthcare and gather data on its efficacy, it is the responsibility of coders, researchers and physicians at every level of the process to educate themselves on this topic and keep open minds to how their work is being communicated to the populations they are serving. Table 2 summarizes key ethical challenges associated with the use of AI in clinical settings, including algorithmic bias, lack of data diversity, transparency issues, and privacy concerns. For each challenge, a representative example is provided along with a proposed solution aimed at promoting responsible and patient-centered AI integration in healthcare systems.

## 6. Case Studies and Emerging Applications

### 6.1. The Growing Need for Innovative Solutions in Resource-Limited U.S. Communities

Although the United States is one of the most technologically advanced and medically resourced nations, many regions across the country continue to experience barriers to healthcare access. According to estimates from the U.S. Department of Health and Human Services, over 100 million people may face challenges in obtaining timely and adequate care. The National Association of Community Health Centers (NACHC) further reports that the number of Americans medically disenfranchised has doubled since 2014 [59]. A primary contributor to these challenges is the persistent shortage of primary care providers. One promising avenue for addressing these gaps is the application of AI- and ML-enhanced mobile health (mHealth) technologies, which can help extend the reach of care delivery, particularly in areas with constrained healthcare infrastructure [59].

### 6.2. Smartphone-Enabled Diagnostics and Personal Health Assistants

AI and ML technologies are increasingly being applied in U.S. health systems for functions such as real-time patient monitoring and triage support in clinical conditions including diabetes, asthma, and sleep apnea [59,60]. AI-enabled smartphone applications and wearable devices—often leveraging embedded cameras and sensors—have demonstrated promise in helping patients make informed decisions while improving remote connectivity to providers [59]. In both rural and low-resource urban areas, mobile triage apps have been associated with reduced unnecessary emergency department visits, and studies show that over 90% of surveyed participants in these areas reported access to a cellular device [61].

As these tools continue to evolve, their integration with FDA-approved AI-powered health assistants may further enhance patients’ ability to navigate care pathways in the absence of immediate access to a physician. Point-of-care diagnostics powered by AI—using smartphone microphones and cameras—have already been explored for conditions such as otitis media, dermatologic screening, and mental health assessments, including detection of depression and suicidal ideation based on voice analysis [59,62]. These advancements offer scalable opportunities to support triage and early intervention, helping ensure that specialist resources are directed to the patients who need them most.

### 6.3. Diabetic Monitoring and AI-Enabled Screening Programs

AI technologies have shown particular effectiveness in the management of chronic conditions such as diabetes. For instance, image-based AI tools have demonstrated value in monitoring diabetic foot ulcers by identifying early signs of complications and transmitting alerts to healthcare providers, thereby enabling timely interventions [63]. This capability not only improves outcomes but may also reduce hospital admissions and overall healthcare burden, particularly in facilities already impacted by staffing shortages.

In addition, AI/ML-based screening for diabetic retinopathy has emerged as a scalable and effective solution. FDA-approved AI platforms now analyze retinal images with high sensitivity and specificity, enabling early detection of diabetic eye disease without requiring an ophthalmologist on-site. These systems have been successfully deployed in community health clinics and national screening programs, particularly in rural or resource-limited areas. By triaging patients who require specialist attention and identifying those who can be safely monitored remotely, these tools help optimize referral pathways and improve allocation of limited clinical resources [63].

AI-supported screening for diabetic retinopathy is another area of success. FDA-approved algorithms can now evaluate retinal images for early signs of disease without the need for on-site ophthalmologists. These tools have been implemented in community-based settings and national screening programs, particularly benefiting areas where access to specialists is limited. By stratifying patients based on risk, AI tools help prioritize in-person care where it is most needed, supporting better allocation of healthcare resources [63]. These innovations illustrate how AI can support chronic disease management by enhancing clinical efficiency and supporting earlier intervention. In doing so, they help reinforce care systems in regions facing persistent constraints in workforce and infrastructure [63].

### 6.4. Community-Level Applications of AI in Public Health

Beyond individual care, AI technologies can also contribute to population-level initiatives. In primary care settings, clinical decision support systems incorporating AI have been developed to guide treatment planning based on peer-reviewed evidence and clinical trial data [60,63,64]. Public health programs may also benefit from AI applications originally developed for other industries, such as finance and marketing, where predictive algorithms are used to analyze trends and forecast outcomes.

These tools could be leveraged by community health initiatives to detect emerging health risks, optimize the use of limited outreach resources, and improve programmatic planning. By engaging patients who may otherwise delay or avoid care, these applications may help enhance participation in community-based health initiatives, supporting improved access and care continuity [59].

### 6.5. Considerations for Algorithm Design and Implementation

Despite the promise of AI, effective integration into healthcare delivery systems requires thoughtful consideration of model design and training. Algorithms developed using non-representative datasets can yield biased outputs and affect clinical decision-making. One review demonstrated that certain AI-based risk prediction tools underestimated illness severity in African American patients due to training data limitations [65]. However, the same study noted that algorithmic bias could be mitigated by incorporating diverse and representative datasets.

In addition, historically underrepresented populations—including those from low-income areas—have often been excluded from research data, further complicating AI development [64,66]. For AI tools to serve their intended users effectively, developers must intentionally design and validate algorithms using comprehensive datasets. Parallel to technical development, there is a need to ensure community readiness through education, engagement, and the cultivation of trust among patients and providers alike.

### 6.6. Navigating Privacy, Regulation, and Infrastructure Needs

As AI technologies gain traction in healthcare, concerns surrounding regulation and patient privacy have become increasingly important. AI-enabled tools often rely on the collection and transmission of sensitive health data, raising questions around compliance with standards like the Health Insurance Portability and Accountability Act. The rapid evolution of the AI landscape has outpaced current regulatory frameworks, underscoring the need for oversight mechanisms to ensure patient confidentiality, algorithmic transparency, and responsible data stewardship [60,63,64].

### 6.7. Conclusion: Realizing the Potential of AI in Expanding Access to Immunotherapy

Taken together, the tools and systems described in this review demonstrate the capacity of AI technologies to enhance healthcare delivery, especially through mobile health platforms, diagnostic innovations, and clinical decision support. In resource-limited communities across the United States, these technologies offer scalable approaches to expanding access, improving chronic disease management, and optimizing use of limited clinical personnel.

As AI continues to evolve, its integration into healthcare initiatives holds great promise for supporting the delivery of advanced therapies, including immunotherapy. By increasing efficiency, supporting early intervention, and enabling care delivery across diverse geographic and clinical settings, AI-driven solutions may help transform cancer care pathways and bring high-quality services within reach for more communities than ever before.

## 7. Research Gaps and Future Directions

Despite promising developments in AI-enhanced cancer immunotherapy, significant research gaps remain, particularly in the unbiased implementation and effectiveness of these technologies for underserved populations. Addressing these gaps is essential to ensure that AI solutions do not perpetuate existing challenges but act as a tool to improve access to inclusive cancer care.

### 7.1. Lack of Diverse and Representative Datasets

One of the most critical research gaps in AI oncology research is data standardization and quality. Most AI models in oncology are trained on datasets derived from academic medical centers and clinical trials, which often underrepresent minority and low-income populations [66]. Several barriers to healthcare research exist for minority populations, including mistrust and fear of participation, stigma associated with research involvement, and competing demands [67]. Despite minorities making up 30% of the U.S. population, they represent fewer than 18% of participants in the National Cancer Institute’s publicly funded cancer clinical trials [67]. Due to these barriers, AI models may perform suboptimal when applied to diverse patient populations, increasing the risk of algorithmic bias and leading to disparities in diagnosis and treatment recommendations [66]. A scoping review by D’Amiano et al. of 118 AI studies in oncology revealed that only 5% reported racial demographics of participants in their training or validation datasets, with 87.8% of reported participants identified as White, highlighting a significant lack of diversity in datasets used for AI model development [68].

### 7.2. Technical Challenges in Multimodal Data Integration

Additionally, AI applications in oncology depend significantly on extracting and analyzing complex, multimodal data, including medical images, genetic profiles, and unstructured clinical notes [69]. This complexity necessitates advanced computational approaches like deep learning and natural language processing to effectively interpret and integrate these diverse data types [70]. However, many current cancer biomarkers remain unimodal, limiting their clinical potential [69]. For instance, glioma patients with similar genetic or histological features may have varying outcomes due to macroscopic factors such as tumor location limiting surgical resection and effective radiation or disruption of the blood–brain barrier, affecting drug delivery efficacy [71]. Chen et al. analyzed data from 5720 patients across 14 cancer types, demonstrating that a deep learning model integrating whole-slide histology images with genetic and molecular data improved risk stratification in 9 of 14 cancer types compared to models using single data types [72]. Similarly, Mahootiha et al. showed that multimodal models outperformed single-modality models in predicting event-free survival among pediatric patients with low-grade gliomas but also highlighted several persistent limitations [73]. For instance, combining data from multiple institutions introduced variability due to differences in imaging protocols, clinical practices, and data annotation standards, which impacted model reliability [67]. Additionally, the authors observed reduced model performance when models trained on data from a single institution were generalized to broader populations, emphasizing challenges in achieving consistent performance across diverse clinical settings [73]. Addressing these technical challenges is crucial for fully realizing AI’s potential in oncology, particularly in ensuring robust performance across diverse clinical settings.

### 7.3. Limited Clinical Integration and Implementation Science

Implementation science is another critical area needing focused study. Although proof of-concept AI models are increasingly common, their integration into clinical workflows remains limited due to physician concerns and insufficient clinical evidence. A systematic review by Macheka et al. identified key barriers to implementing AI in routine oncology practice, noting that most AI research remains experimental, with limited prospective clinical validation and minimal evidence translating efficacy into meaningful clinical outcomes [74]. Current literature predominantly emphasizes the technical feasibility of AI applications in oncology, with limited attention to critical clinical outcomes such as progression-free survival, adverse event rates, and access to treatment across demographic groups [74,75]. Future research must prioritize these endpoints to ensure AI tools effectively contribute to improved patient outcomes and lessening existing barriers to accessing care.

### 7.4. Promising Models of Interdisciplinary Collaboration

Collaboration among AI researchers, clinicians, and community health workers has demonstrated promising results in enhancing cancer care, particularly by addressing disparities and improving health outcomes. For instance, a community-based lung cancer screening initiative in rural Eastern China effectively combined mobile low-dose computed tomography (LDCT) units with AI technologies. This collaborative approach integrated the expertise of community health workers, nurses, physicians, and patient navigators, resulting in a feasible and cost-effective strategy that significantly reduced health disparities in underserved rural populations [76]. Additionally, multidisciplinary partnerships between AI researchers and oncology teams have successfully developed multimodal deep learning models, improved recurrence risk prediction and enabling personalized treatment strategies for pediatric glioma patients [73]. Public–private collaborations have further advanced impactful AI implementations. For example, a study by Abreu et al. (2024) illustrated that utilizing OpenAI technologies to rewrite cancer-related patient education materials significantly enhanced readability, transitioning from a university freshman to a high school freshman reading level, without compromising content quality. This approach holds substantial promise for increasing cancer literacy and reducing health challenges by ensuring that crucial health information is accessible to patients across diverse literacy levels [11].

### 7.5. Future Priorities for Objective AI Integration

Collectively, these examples highlight the significant potential of interdisciplinary collaboration in advancing the practical applicability, effectiveness, and rational AI-driven cancer care. Future initiatives should build on these successes, fostering continued collaboration among researchers, clinicians, and community health workers to design, validate, and implement AI solutions specifically tailored to meet the needs of underserved communities.

## 8. Conclusions

Importantly, AI tools are not only enhancing provider efficiency but also addressing critical challenges related to geographic isolation, limited healthcare workforce capacity, and uneven distribution of specialist services. In doing so, they are playing a growing role in closing longstanding gaps in care for populations. The capacity to scale these tools across a wide range of healthcare settings—from large academic centers to community clinics—underscores their potential to support more consistent, standardized, and high-quality care delivery, regardless of a patient’s location or socioeconomic status.

However, the promise of AI must be matched by a commitment to ethical, equitable implementation. This includes ensuring that AI algorithms are trained on diverse and representative datasets to prevent perpetuation of bias, as well as promoting transparency in how AI-derived recommendations are made. Digital literacy, access to broadband infrastructure, and culturally appropriate interfaces must also be considered, particularly when engaging historically underserved populations. Furthermore, building trust among patients and providers through education, regulation, and oversight will be essential for widespread adoption.

Sustainable success will also require interdisciplinary collaboration between healthcare professionals, data scientists and public health experts. Incentivizing AI innovation through funding, while simultaneously establishing clear regulatory frameworks, can help ensure that these technologies are both cutting-edge and responsibly governed. Moreover, incorporating the voices of patients and community stakeholders in the design and deployment of AI-driven tools will enhance their usability, relevance, and effectiveness.

**Figure 2:**
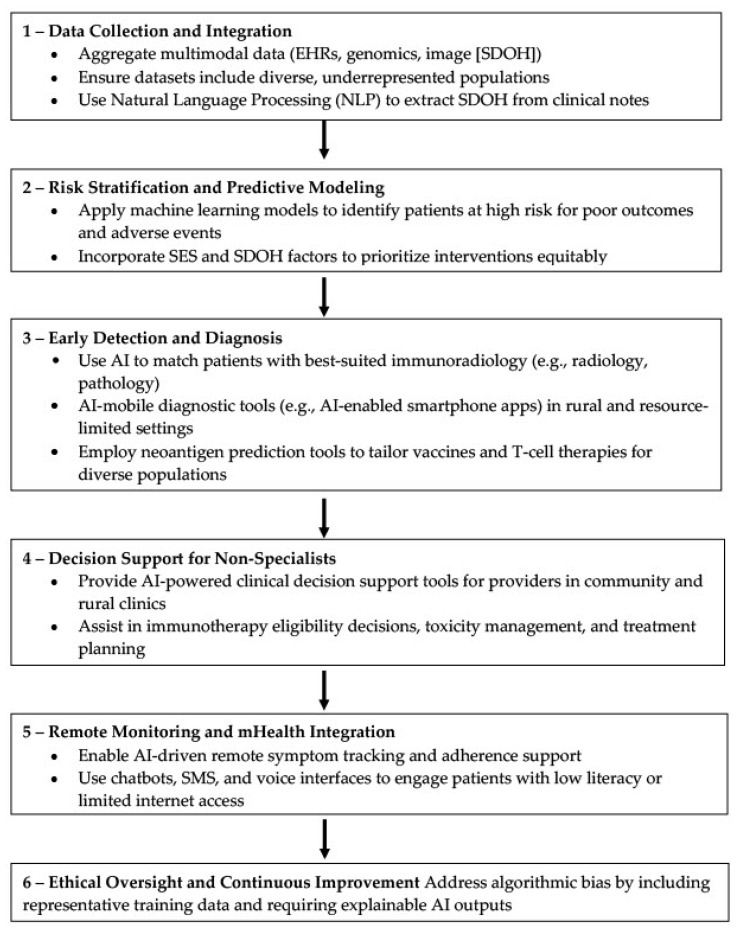
AI-driven algorithm to improve immunotherapy accessibility in underserved communities. This flowchart presents a six-step AI framework—ranging from data integration and risk stratification to AI-enhanced diagnosis, decision support, remote monitoring, and ethical oversight—designed to reduce disparities and enhance delivery of advanced immunotherapy. The model emphasizes equitable data use, clinical support for non-specialists, patient engagement in low-resource settings, and transparent, bias-aware implementation.

In sum, AI should be seen not as a substitute for human care, but as a critical enabler that can amplify the capabilities of healthcare systems and professionals. When thoughtfully integrated, AI technologies can reduce administrative burden, increase diagnostic reach, and facilitate earlier, more personalized interventions as demonstrated in Figure 2. For cancer care, chronic disease management, and beyond, the strategic application of AI holds the potential to reimagine health systems—bringing us closer to a future where high-quality care is not a privilege, but a universal standard.

## Figures and Tables

**Table 1:** Comparative Summary of AI Technologies and Models in Cancer Immunotherapy and Their Impact on Health Disparities.

AI Tool/Model	Application	Key Features	Performance Metrics	Impact on Disparities
Natural Language Processing (NLP)	Extracting clinical notes, chatbot interfaces	Multilingual processing, unstructured data analysis	Context-specific; varies by implementation	Improves access via multilingual support and outreach to diverse populations
Machine Learning	Disease diagnosis, outcome prediction	Supervised/unsupervised learning, pattern recognition	Accuracy: variable; e.g., AUC > 0.80 in some cases	Can reduce care variability; risks perpetuating bias if training data lacks representation
Predictive Analytics	Risk stratification, resource allocation	Statistical modeling with clinical + social data	ROC AUC: 0.75–0.90 typically	Enables proactive interventions when SDOH data are included
Computer Vision	Radiology, histopathology, dermatology	CNNs, image segmentation and classification	AUC > 0.90 in radiology/dermatology	May underperform on darker skin tones if training datasets lack diversity
Recommendation Algorithms	Clinical decision support, personalized treatments	Knowledge graphs, EHR integration	Concordance with clinicians: 70–90%	Can promote equity in care delivery; risks disparities without transparency
Speech Recognition	Patient interaction, low-literacy communication	Voice-to-text AI, language understanding	Word error rate varies by accent/language	Enhances access for patients with low literacy or physical disabilities
PathAI	Histopathology image analysis	CNN-based H&E tissue interpretation	AUC: 0.93 (breast cancer); accuracy: ~90%	Limited demographic testing; currently focused on TCGA datasets
DeepSurv	Prognostic modeling	Deep neural Cox model for survival prediction	C-index: 0.74–0.82	Limited validation in underserved populations
Tempus xT Platform	Genomic profiling + therapy response	AI-driven NGS + clinical decision support	Clinical utility: ~85% actionable findings	Limited data from diverse or global cohorts
Lunit SCOPE IO	Immune microenvironment profiling	AI-based spatial tissue analysis	AUC: 0.89 (NSCLC); specificity: 88%	Needs broader population testing

Table 1: Examples of AI Tools and Their Applications. This table presents various AI technologies, their clinical and operational uses, and how they influence healthcare disparities. While many tools offer opportunities to improve access, efficiency, and personalization of care, each also presents unique risks related to bias, data representativeness, and implementation. Equity-focused design and validation are critical for maximizing the benefits of these technologies.

**Table 2:** Ethical Considerations in AI Implementation.

Ethical Challenge	Example	Proposed Solution
Algorithmic Bias	Higher false-negative rates in AI-based skin cancer detection on darker skin tones	Incorporate diverse datasets and perform subgroup performance validation
Data Diversity	Underrepresentation of rural and minority populations in training data	Engage community stakeholders and build inclusive data collection protocols
Transparency	Black-box AI systems in clinical decision-making	Mandate explainable AI tools and clinician education on AI interpretation
Privacy Concerns	Use of patient data without clear consent or anonymization	Establish strong governance frameworks and patient-centered data sharing policies

Table 2: Ethical Considerations in AI Implementation. This table highlights common ethical issues that arise during the development and implementation of AI tools in healthcare. It provides real-world examples of each challenge and outlines actionable solutions to mitigate risks related to bias, inclusivity, transparency, and patient privacy. These strategies aim to ensure ethical and trustworthy use of AI technologies in clinical practice.

## References

[1] Kumar A.R., Devan A.R., Nair B., Vinod B.S., Nath L.R. (2021). Harnessing the Immune System against Cancer: Current Immunotherapy Approaches and Therapeutic Targets. Mol. Biol. Rep..

[2] Sordo-Bahamonde C., Lorenzo-Herrero S., Gonzalez-Rodriguez A.P., Martínez-Pérez A., Rodrigo J.P., García-Pedrero J.M., Gonzalez S. (2023). Chemo-Immunotherapy: A New Trend in Cancer Treatment. Cancers.

[3] Hosseinkhani N., Derakhshani A., Kooshkaki O., Abdoli Shadbad M., Hajiasgharzadeh K., Baghbanzadeh A., Safarpour H., Mokhtarzadeh A., Brunetti O., Yue S.C. (2020). Immune Checkpoints and CAR-T Cells: The Pioneers in Future Cancer Therapies?. Int. J. Mol. Sci..

[4] Yarchoan M., Gane E.J., Marron T.U., Perales-Linares R., Yan J., Cooch N., Shu D.H., Fertig E.J., Kagohara L.T., Bartha G. (2024). Personalized Neoantigen Vaccine and Pembrolizumab in Advanced Hepatocellular Carcinoma: A Phase 1/2 Trial. Nat. Med..

[5] Gargett T., Truong N.T.H., Gardam B., Yu W., Ebert L.M., Johnson A., Yeo E.C.F., Wittwer N.L., Tapia Rico G., Logan J. (2024). Safety and Biological Outcomes Following a Phase 1 Trial of GD2-Specific CAR-T Cells in Patients with GD2-Positive Metastatic Melanoma and Other Solid Cancers. J. Immunother. Cancer.

[6] Chen M., Sun R., Shi B., Wang Y., Di S., Luo H., Sun Y., Li Z., Zhou M., Jiang H. (2019). Antitumor Efficacy of Chimeric Antigen Receptor T Cells against EGFRvIII-Expressing Glioblastoma in C57BL/6 Mice. Biomed. Pharmacother. Biomed. Pharmacother..

[7] Li H., Ding J., Lu M., Liu H., Miao Y., Li L., Wang G., Zheng J., Pei D., Zhang Q. (2020). CAIX-Specific CAR-T Cells and Sunitinib Show Synergistic Effects Against Metastatic Renal Cancer Models. J. Immunother..

[8] Winkfield K.M., Regnante J.M., Miller-Sonet E., González E.T., Freund K.M., Doykos P.M. (2021). Development of an Actionable Framework to Address Cancer Care Disparities in Medically Underserved Populations in the United States: Expert Roundtable Recommendations. JCO Oncol. Pract..

[9] Liang J., Wolsiefer K., Zestcott C.A., Chase D., Stone J. (2019). Implicit Bias toward Cervical Cancer: Provider and Training Differences. Gynecol. Oncol..

[10] Schatz A.A., Brooks-Coley K., Harrington E., Murray M.S., Carlson R.W. (2022). Patient, Caregiver, and Oncologist Experiences with and Perceptions of Racial Bias and Discrimination in Cancer Care Delivery. J. Natl. Compr. Cancer Netw. JNCCN.

[11] Abreu A.A., Murimwa G.Z., Farah E., Stewart J.W., Zhang L., Rodriguez J., Sweetenham J., Zeh H.J., Wang S.C., Polanco P.M. (2024). Enhancing Readability of Online Patient-Facing Content: The Role of AI Chatbots in Improving Cancer Information Accessibility. J. Natl. Compr. Cancer Netw. JNCCN.

[12] Xiong S., Fu Z., Deng Z., Li S., Zhan X., Zheng F., Yang H., Liu X., Xu S., Liu H. (2024). Machine Learning-Based CT Radiomics Enhances Bladder Cancer Staging Predictions: A Comparative Study of Clinical, Radiomics, and Combined Models. Med. Phys..

[13] Zhang Z., Cao B., Wu J., Feng C. (2024). Development and Validation of an Interpretable Machine Learning Prediction Model for Total Pathological Complete Response after Neoadjuvant Chemotherapy in Locally Advanced Breast Cancer: Multicenter Retrospective Analysis. J. Cancer.

[14] Montoya C., Spieler B., Welford S.M., Kwon D., Pra A.D., Lopes G., Mihaylov I.B. (2023). Predicting Response to Immunotherapy in Non-Small Cell Lung Cancer- from Bench to Bedside. Front. Oncol..

[15] Mørk S.K., Kadivar M., Bol K.F., Draghi A., Westergaard M.C.W., Skadborg S.K., Overgaard N., Sørensen A.B., Rasmussen I.S., Andreasen L.V. (2022). Personalized Therapy with Peptide-Based Neoantigen Vaccine (EVX-01) Including a Novel Adjuvant, CAF®09b, in Patients with Metastatic Melanoma. Oncoimmunology.

[16] Li J., Yuan Y., Bian L., Lin Q., Yang H., Ma L., Xin L., Li F., Zhang S., Wang T. (2023). A Comparison between Clinical Decision Support System and Clinicians in Breast Cancer. Heliyon.

[17] Verdini N.P., Bryl K.L., Baser R.E., Lapen K., Mao J.J., Gillespie E.F. (2024). Patient-Reported Outcomes as a Recruitment Strategy for Clinical Trial Enrollment. JAMA Oncol..

[18] Parikh R.B., Guido M., Girard A., Li Y., Kolla L., Chen J., Emanuel E.J. (2024). Human-AI Teams to Improve Accuracy and Timeliness of Oncology Trial Prescreening: Preplanned Interim Analysis of a Randomized Trial. J. Clin. Oncol..

[19] Da’Costa A., Teke J., Origbo J.E., Osonuga A., Egbon E., Olawade D.B. (2025). AI-Driven Triage in Emergency Departments: A Review of Benefits, Challenges, and Future Directions. Int. J. Med. Inf..

[20] Tyler S., Olis M., Aust N., Patel L., Simon L., Triantafyllidis C., Patel V., Lee D.W., Ginsberg B., Ahmad H. (2024). Use of Artificial Intelligence in Triage in Hospital Emergency Departments: A Scoping Review. Cureus.

[21] Blue B. (2022). Socioeconomic and Racial Disparity in Chimeric Antigen Receptor T Cell (CART)Therapy Access. Transplant. Cell. Ther..

[22] Fiscella K., Sanders M.R. (2016). Racial and Ethnic Disparities in the Quality of Health Care. Annu. Rev. Public Health.

[23] Naylor K.B., Tootoo J., Yakusheva O., Shipman S.A., Bynum J.P.W., Davis M.A. (2019). Geographic Variation in Spatial Accessibility of U.S. Healthcare Providers. PLoS ONE.

[24] Syed S.T., Gerber B.S., Sharp L.K. (2013). Traveling towards Disease: Transportation Barriers to Health Care Access. J. Community Health.

[25] Heiat A., Gross C.P., Krumholz H.M. (2002). Representation of the Elderly, Women, and Minorities in Heart Failure Clinical Trials. Arch. Intern. Med..

[26] Duma N., Vera Aguilera J., Paludo J., Haddox C.L., Gonzalez Velez M., Wang Y., Leventakos K., Hubbard J.M., Mansfield A.S., Go R.S. (2018). Representation of Minorities and Women in Oncology Clinical Trials: Review of the Past 14 Years. J. Oncol. Pract..

[27] Kwiatkowski K., Coe K., Bailar J.C., Swanson G.M. (2013). Inclusion of Minorities and Women in Cancer Clinical Trials, a Decade Later: Have We Improved?. Cancer.

[28] Abbott E.E., Apakama D., Richardson L.D., Chan L., Nadkarni G.N. (2024). Leveraging Artificial Intelligence and Data Science for Integration of Social Determinants of Health in Emergency Medicine: Scoping Review. JMIR Med. Inform..

[29] Juhn Y.J., Ryu E., Wi C.-I., King K.S., Malik M., Romero-Brufau S., Weng C., Sohn S., Sharp R.R., Halamka J.D. (2022). Assessing Socioeconomic Bias in Machine Learning Algorithms in Health Care: A Case Study of the HOUSES Index. J. Am. Med. Inform. Assoc. JAMIA.

[30] Carroll N.W., Jones A., Burkard T., Lulias C., Severson K., Posa T. (2022). Improving Risk Stratification Using AI and Social Determinants of Health. Am. J. Manag. Care.

[31] Müller M., Huber F., Arnaud M., Kraemer A.I., Altimiras E.R., Michaux J., Taillandier-Coindard M., Chiffelle J., Murgues B., Gehret T. (2023). Machine Learning Methods and Harmonized Datasets Improve Immunogenic Neoantigen Prediction. Immunity.

[32] Li T., Li Y., Zhu X., He Y., Wu Y., Ying T., Xie Z. (2023). Artificial Intelligence in Cancer Immunotherapy: Applications in Neoantigen Recognition, Antibody Design and Immunotherapy Response Prediction. Semin. Cancer Biol..

[33] Cai Y., Chen R., Gao S., Li W., Liu Y., Su G., Song M., Jiang M., Jiang C., Zhang X. (2022). Artificial Intelligence Applied in Neoantigen Identification Facilitates Personalized Cancer Immunotherapy. Front. Oncol..

[34] Eysenbach G. (2023). The Role of ChatGPT, Generative Language Models, and Artificial Intelligence in Medical Education: A Conversation with ChatGPT and a Call for Papers. JMIR Med. Educ..

[35] Al Shamsi H., Almutairi A.G., Al Mashrafi S., Al Kalbani T. (2020). Implications of Language Barriers for Healthcare: A Systematic Review. Oman Med. J..

[36] Fatima A., Shafique M.A., Alam K., Fadlalla Ahmed T.K., Mustafa M.S. (2024). ChatGPT in Medicine: A Cross-Disciplinary Systematic Review of ChatGPT’s (Artificial Intelligence) Role in Research, Clinical Practice, Education, and Patient Interaction. Medicine.

[37] Morrow E., Zidaru T., Ross F., Mason C., Patel K.D., Ream M., Stockley R. (2023). Artificial Intelligence Technologies and Compassion in Healthcare: A Systematic Scoping Review. Front. Psychol..

[38] Milne-Ives M., De Cock C., Lim E., Shehadeh M.H., De Pennington N., Mole G., Normando E., Meinert E. (2020). The Effectiveness of Artificial Intelligence Conversational Agents in Health Care: Systematic Review. J. Med. Internet Res..

[39] Klann J.G., Szolovits P. (2009). An Intelligent Listening Framework for Capturing Encounter Notes from a Doctor-Patient Dialog. BMC Med. Inform. Decis. Mak..

[40] Deliberato R.O., Celi L.A., Stone D.J. (2017). Clinical Note Creation, Binning, and Artificial Intelligence. JMIR Med. Inform..

[41] Peterson Health Technology Institute AI Taskforce (2025). Adoption of Artificial Intelligence in Healthcare Delivery Systems: Early Applications and Impacts.

[42] Lee C., Britto S., Diwan K. (2024). Evaluating the Impact of Artificial Intelligence (AI) on Clinical Documentation Efficiency and Accuracy Across Clinical Settings: A Scoping Review. Cureus.

[43] Goss F.R., Blackley S.V., Ortega C.A., Kowalski L.T., Landman A.B., Lin C.-T., Meteer M., Bakes S., Gradwohl S.C., Bates D.W. (2019). A Clinician Survey of Using Speech Recognition for Clinical Documentation in the Electronic Health Record. Int. J. Med. Inf..

[44] Kernberg A., Gold J.A., Mohan V. (2024). Using ChatGPT-4 to Create Structured Medical Notes From Audio Recordings of Physician-Patient Encounters: Comparative Study. J. Med. Internet Res..

[45] Toole J., Kohansieh M., Khan U., Romero S., Ghali M., Zeltser R., Makaryus A.N. (2020). Does Your Patient Understand Their Treatment Plan? Factors Affecting Patient Understanding of Their Medical Care Treatment Plan in the Inpatient Setting. J. Patient Exp..

[46] Jin Q., Wang Z., Floudas C.S., Chen F., Gong C., Bracken-Clarke D., Xue E., Yang Y., Sun J., Lu Z. (2024). Matching Patients to Clinical Trials with Large Language Models. Nat. Commun..

[47] Calaprice-Whitty D., Galil K., Salloum W., Zariv A., Jimenez B. (2020). Improving Clinical Trial Participant Prescreening with Artificial Intelligence (AI): A Comparison of the Results of AI-Assisted vs Standard Methods in 3 Oncology Trials. Ther. Innov. Regul. Sci..

[48] Rajwal S., Zhang Z., Chen Y., Rogers H., Sarker A., Xiao Y. (2025). Applications of Natural Language Processing and Large Language Models for Social Determinants of Health: Protocol for a Systematic Review. JMIR Res. Protoc..

[49] Jerfy A., Selden O., Balkrishnan R. (2024). The Growing Impact of Natural Language Processing in Healthcare and Public Health. Inq. J. Med. Care Organ. Provis. Financ..

[50] Kelley K. (2024). How Does AI Work? A Beginner’s Guide.

[51] Jain R., Singh M., Rao A.R., Garg R. (2024). Predicting Hospital Length of Stay Using Machine Learning on a Large Open Health Dataset. BMC Health Serv. Res..

[52] Symum H., Zayas-Castro J.L. (2020). Prediction of Chronic Disease-Related Inpatient Prolonged Length of Stay Using Machine Learning Algorithms. Healthc. Inform. Res..

[53] Nordling L. (2019). A Fairer Way Forward for AI in Health Care. Nature.

[54] Álvarez-Machancoses Ó., DeAndrés Galiana E.J., Cernea A., Fernández Sánchez De La Viña J., Fernández-Martínez J.L. (2020). On the Role of Artificial Intelligence in Genomics to Enhance Precision Medicine. Pharmacogenom. Pers. Med..

[55] Duong D., Solomon B.D. (2025). Artificial Intelligence in Clinical Genetics. Eur. J. Hum. Genet..

[56] U.S. Department of Health and Human Services (2025). Summary of the HIPAA Privacy Rule.

[57] Vilhekar R.S., Rawekar A. (2024). Artificial Intelligence in Genetics. Cureus.

[58] Sullivan H.R., Schweikart S.J. (2019). Are Current Tort Liability Doctrines Adequate for Addressing Injury Caused by AI?. AMA J. Ethics.

[59] Bhatt P., Liu J., Gong Y., Wang J., Guo Y. (2022). Emerging Artificial Intelligence-Empowered mHealth: Scoping Review. JMIR mHealth uHealth.

[60] Maleki Varnosfaderani S., Forouzanfar M. (2024). The Role of AI in Hospitals and Clinics: Transforming Healthcare in the 21st Century. Bioengineering.

[61] van Veen T., Binz S., Muminovic M., Chaudhry K., Rose K., Calo S., Rammal J.-A., France J., Miller J.B. (2019). Potential of Mobile Health Technology to Reduce Health Disparities in Underserved Communities. West. J. Emerg. Med..

[62] Topol E.J. (2019). High-Performance Medicine: The Convergence of Human and Artificial Intelligence. Nat. Med..

[63] Mackenzie S.C., Sainsbury C.A.R., Wake D.J. (2024). Diabetes and Artificial Intelligence beyond the Closed Loop: A Review of the Landscape, Promise and Challenges. Diabetologia.

[64] Bajwa J., Munir U., Nori A., Williams B. (2021). Artificial Intelligence in Healthcare: Transforming the Practice of Medicine. Future Healthc. J..

[65] d’Elia A., Gabbay M., Rodgers S., Kierans C., Jones E., Durrani I., Thomas A., Frith L. (2022). Artificial Intelligence and Health Inequities in Primary Care: A Systematic Scoping Review and Framework. Fam. Med. Community Health.

[66] Green B.L., Murphy A., Robinson E. (2024). Accelerating Health Disparities Research with Artificial Intelligence. Front. Digit. Health.

[67] George S., Duran N., Norris K. (2014). A Systematic Review of Barriers and Facilitators to Minority Research Participation among African Americans, Latinos, Asian Americans, and Pacific Islanders. Am. J. Public Health.

[68] D’Amiano A.J., Cheunkarndee T., Azoba C., Chen K.Y., Mak R.H., Perni S. (2025). Transparency and Representation in Clinical Research Utilizing Artificial Intelligence in Oncology: A Scoping Review. Cancer Med..

[69] Lipkova J., Chen R.J., Chen B., Lu M.Y., Barbieri M., Shao D., Vaidya A.J., Chen C., Zhuang L., Williamson D.F.K. (2022). Artificial Intelligence for Multimodal Data Integration in Oncology. Cancer Cell.

[70] Sun Y., Song N., Han Y., Ding S. (2023). Organic Base-Facilitated Thiol-Thioalkyne Reaction with Exclusive Regio- and Stereoselectivity. J. Org. Chem..

[71] Miller G. (2002). Drug Targeting. Breaking down Barriers. Science.

[72] Chen R.J., Lu M.Y., Williamson D.F.K., Chen T.Y., Lipkova J., Noor Z., Shaban M., Shady M., Williams M., Joo B. (2022). Pan-Cancer Integrative Histology-Genomic Analysis via Multimodal Deep Learning. Cancer Cell.

[73] Mahootiha M., Tak D., Ye Z., Zapaishchykova A., Likitlersuang J., Climent Pardo J.C., Boyd A., Vajapeyam S., Chopra R., Prabhu S.P. (2025). Multimodal Deep Learning Improves Recurrence Risk Prediction in Pediatric Low-Grade Gliomas. Neuro-Oncol..

[74] Macheka S., Ng P.Y., Ginsburg O., Hope A., Sullivan R., Aggarwal A. (2024). Prospective Evaluation of Artificial Intelligence (AI) Applications for Use in Cancer Pathways Following Diagnosis: A Systematic Review. BMJ Oncol..

[75] Taylor M., Liu X., Denniston A., Esteva A., Ko J., Daneshjou R., Chan A.-W. (2021). SPIRIT-AI and CONSORT-AI Working Group Raising the Bar for Randomized Trials Involving Artificial Intelligence: The SPIRIT-Artificial Intelligence and CONSORT-Artificial Intelligence Guidelines. J. Investig. Dermatol..

[76] Huang F., Lin X., Hong Y., Li Y., Li Y., Chen W.-T., Chen W. (2025). The Feasibility and Cost-Effectiveness of Implementing Mobile Low-Dose Computed Tomography with an AI-Based Diagnostic System in Underserved Populations. BMC Cancer.

